# Rapid Colorimetric Detection of Genome Evolution in SCRaMbLEd Synthetic *Saccharomyces cerevisiae* Strains

**DOI:** 10.3390/microorganisms8121914

**Published:** 2020-12-01

**Authors:** Elizabeth L. I. Wightman, Heinrich Kroukamp, Isak S. Pretorius, Ian T. Paulsen, Helena K. M. Nevalainen

**Affiliations:** 1 Centre of Excellence in Synthetic Biology, Department of Molecular Sciences, Macquarie University, Sydney, NSW 2109, Australia; Elizabeth.Wightman@HDR.mq.edu.au (E.L.I.W.); Ian.Paulsen@mq.edu.au (I.T.P.); Helena.Nevalainen@mq.edu.au (H.K.M.N.); 2 Biomolecular Discovery and Design Research Centre, Macquarie University, Sydney, NSW 2109, Australia; 3 Chancellery, Macquarie University, Sydney, NSW 2109, Australia; Sakkie.Pretorius@mq.edu.au

**Keywords:** *Saccharomyces cerevisiae*, SCRaMbLE, genome evolution, industrial yeast strains

## Abstract

Genome-scale engineering and custom synthetic genomes are reshaping the next generation of industrial yeast strains. The Cre-recombinase-mediated chromosomal rearrangement mechanism of designer synthetic *Saccharomyces cerevisiae* chromosomes, known as SCRaMbLE, is a powerful tool which allows rapid genome evolution upon command. This system is able to generate millions of novel genomes with potential valuable phenotypes, but the excessive loss of essential genes often results in poor growth or even the death of cells with useful phenotypes. In this study we expanded the versatility of SCRaMbLE to industrial strains, and evaluated different control measures to optimize genomic rearrangement, whilst limiting cell death. To achieve this, we have developed RED (rapid evolution detection), a simple colorimetric plate-assay procedure to rapidly quantify the degree of genomic rearrangements within a post-SCRaMbLE yeast population. RED-enabled semi-synthetic strains were mated with the haploid progeny of industrial yeast strains to produce stress-tolerant heterozygous diploid strains. Analysis of these heterozygous strains with the RED-assay, genome sequencing and custom bioinformatics scripts demonstrated a correlation between RED-assay frequencies and physical genomic rearrangements. Here we show that RED is a fast and effective method to evaluate the optimal SCRaMbLE induction times of different Cre-recombinase expression systems for the development of industrial strains.

## 1. Introduction

Specialized strains of the yeast *Saccharomyces cerevisiae* are harnessed by industry for the production of food and beverages, pharmaceuticals, chemical building blocks and fuel. While past strategies such as ALE (adaptive laboratory evolution), random mutagenesis and rational design approaches have produced a myriad of improved strains, the field of genetic engineering now benefits from whole-genome reengineering approaches and the synthesis of custom designer genomes [1]. SCRaMbLE (synthetic chromosome rearrangement and modification by LoxPSym-mediated evolution) is a genome rearrangement system developed for use in the *Saccharomyces cerevisiae* 2.0 (Sc2.0) synthetic genome, facilitating large-scale genomic rearrangements [2]. The system allows gene deletions, insertions, inversion and translocations genome-wide to generate large libraries of unique strains that can be screened for desired phenotypes. 

Valuable industrial characteristics, such as inhibitor tolerance and high protein secretion, often have complex and/or unknown genetic determinants [3,4,5]. Utilizing the genome-scale randomization of SCRaMbLE, improvements of complex phenotypes have already been accomplished [6,7,8,9]. An example of the capability for genome rearrangement was the rapid generation of semi-synthetic heterozygous diploid strains containing a single copy of the synthetic chromosomes synV and synX, with significantly improved thermotolerance at 42 °C after a single round of SCRaMbLE [9], whereas a similar increase in thermotolerance using ALE took over 300 generations [10]. SCRaMbLE was also used to optimize the biosynthetic pathway for improved violacein yields, demonstrating that this strategy could potentially be applied to optimize the production of any metabolite [7]. These SCRaMbLEd strains contained only one synthetic chromosome in a haploid genome context; therefore, it is conceivable that strains harboring more synthetic DNA, with more loxP recombinase recognition sites, could produce greater genomic diversity with associated novel phenotypes of interest. Thus far, SCRaMbLE has also been explored for a variety of fundamental and applied applications, including genome minimization [2], biosynthetic pathway assembly [8,11] and for the optimization of hydrolytic enzyme ratios for biofuel applications [12].

SCRaMbLE has been successfully employed to develop yeast strains with novel phenotypes; however, this is accompanied by the rapid loss of essential genes, especially in haploid cell populations. This often leads to retarded cell growth and high lethality rates, characteristics frequently used to estimate the degree of SCRaMbLE that has occurred in haploid cells [2,13]. In addition to the loss of cell viability, other non-lethal deleterious effects could mask the effects of desirable phenotypes generated through genomic rearrangement, while growth-impaired mutant cells are vulnerable to being outcompeted by healthier, less-SCRaMbLEd cells. It is thus imperative to maintain a balance between the degree of genomic rearrangement and cell viability so as to maximize the discovery of useful phenotypes.

Considering that industrial strains are generally diploid and that the 2n gene copy-number could serve as a viability buffer in the event of essential gene loss, SCRaMbLE holds tremendous potential to augment commercial strain development and improvement [9]. It has previously been shown that the survival of SCRaMbLEd diploids is significantly higher than that of haploids, with a viability over 70% compared to less than 30% in haploid strains under the same treatment conditions [9]. Although SCRaMbLEing in diploid cells overcomes some of the limitations associated with rapid haploid cell death, it abolishes the use of viability as a simple output to gauge the degree of genome scrambling in the population. Simultaneously, it increases the complexity of the bioinformatic analysis of these genomes due to the high sequence similarity between equivalent synthetic and non-synthetic genomic regions. As such, there is a need for new methods which overcome these limitations.

In this study, we have developed RED (rapid evolution detection), a simple colorimetric plate-assay procedure to determine the degree of genomic rearrangements within SCRaMbLEd diploid yeast populations. RED-capable semi-synthetic laboratory strains were combined with haploids from four different industrial strains. The frequency of red-pigmented colonies was quantified and compared to the relative degree of gene loss within randomly selected colonies in the population. We subsequently used the RED system to monitor the leakiness and SCRaMbLE induction profile of several previously reported Cre-recombinase expression vectors. As proof of concept, we have showed that RED could be generally applied to semi-synthetic industrial strains to rapidly evaluate the frequency of genomic rearrangements in a SCRaMbLEd population, which allows the fine-tuning and selection of optimal SCRaMbLE conditions for strain library generation.

## 2. Materials and Methods 

### 2.1. Culturing Media

Chemicals used in this study were obtained from Sigma-Aldrich, Australia and used as recommended by the supplier, unless stated otherwise. *Saccharomyces cerevisiae* strains were routinely cultivated in YPD (10 g L^−1^ *w*/*v* yeast extract, 20 g L^−1^ *w*/*v* peptone, 20 g L^−1^ *w*/*v* glucose) at 30 °C and were shaken at 200 rpm for liquid cultures. *Escherichia coli* DH5α cultures were used for plasmid propagation and were cultivated at 37 °C in Luria Bertani broth or agar (10 g L^−1^ tryptone, 5 g L^−1^ yeast extract, 10 g L^−1^ sodium chloride, 20 g L^−1^ bacteriological agar) supplemented with 100 μg mL^−1^ ampicillin for plasmid selection.

YP-gal agar (10 g L^−1^ yeast extract, 20 g L^−1^ peptone, 20 g L^−1^ galactose), supplemented with 400 μg mL^−1^ hygromycin B (InvivoGen, USA), was used to select *HO-*deleted heterozygous diploid industrial strains and the subsequent haploid progeny. Sporulation agar plates contained 1 g L^−1^ yeast extract, 10 g L^−1^ potassium acetate and 20 g L^−1^ bacteriological agar. 

*S. cerevisiae* strains containing Cre-plasmids were selected and maintained using SC^-ura^ medium (20 g L^−1^ glucose, 1.92 g L^−1^ yeast synthetic drop-out medium supplements without uracil, 0.68 g L^−1^ yeast nitrogen base without amino acids). SC^-ade^ agar (20 g L^−1^ glucose, 0.68 g L^−1^ yeast nitrogen base without amino acids, 20 mg L^−1^ uracil, 20 mg L^−1^ L-methionine, 60 mg L^−1^ L-leucine, 20 mg L^−1^ L-histidine, 20 g L^−1^ bacteriological agar) was used to select for *ADE2*-positive transformants. 

### 2.2. Construction of Cre Plasmids

All DNA manipulations were performed using reagents supplied by New England Biolabs, Australia according to the manufacturer’s recommendation, unless otherwise stated. All PCR primer sequences are provided in Table A1. 

Four plasmids, each containing different native yeast promoters to allow the differential expression of the Cre-recombinase gene, were used in this study (Table 1). The pLM160 plasmid, containing the *CLB2* promoter, has previously been constructed [9,14,15]. To construct pEW_SCW11p, the *SCW11_p_* Cre-EBD cassette was liberated from the pLM006 plasmid by SacI and EcoRI restriction enzyme digestion, and ligated directionally into the corresponding sites of the SacI and EcoRI digested pLM160 plasmid. The *GAL1* promoter from pHK300-HO was isolated by digestion with EcoRI and PacI. The Cre-EBD cassette and the backbone plasmid from pLM160 were PCR-amplified using primers Cre-F+PacI and Cre-CassetteR (Table A1), which added a PacI restriction enzyme recognition site. The PCR amplicon was digested with PacI and EcoRI, and the digested *GAL1* promoter was subsequently ligated into the Cre-EBD containing plasmid backbone, to yield pEW_GAL1p (Table 1).

### 2.3. Industrial Haploid Strain Generation

To generate stable haploid progeny of the industrial strains, *ho*-deletion cassette PCR fragments were generated from the pHK300-HO plasmid (Table 1), using the HO_ampl-F/R primer set (Table A1) and transformed into Y-11878, Y-582, YB-428 and MH-1000 (Table 2) using the LiOAc/SS carrier DNA/PEG transformation method [16], and recovered for 4 h in YP-gal broth before plating onto YP-gal agar, supplemented with 400 μg mL^−1^ G418–sulfate. The galactose-dependent expression of the geneticin resistance gene ensured the minimal influence of its protein product during strain evaluation on glucose-containing media. Putative transformants were selected and the disruption of at least one *HO* allele was confirmed with PCR using the HO_conf-F/R primer set (Table A1). Y-11878, Y-582, YB-428 and MH-1000 strains heterozygous for a functional *HO* gene were grown to the stationary phase in YPD medium. A thick cell suspension of each culture was spread on sporulation agar plates and incubated at ambient temperature until sufficient asci formation was observed (after 3–6 days). Random spore isolation was performed as previously described [5] and spore suspensions were plated on YP-gal agar, supplemented with 400 μg mL^−1^ hygromycin B to select for *HO-*disrupted haploid progeny. The *HO* gene encodes an endonuclease which allows yeast to convert between mating types, thus facilitating spontaneous diploid formation. The colonies were selected form the MH-1000, Y-11878, YB-428 and Y-582 backgrounds and were designated HK01, HK02, HK03 and HK04, respectively. The mating type selection was performed by multiplex PCR using the MatLocus, MatA and MatAlpha primers (Table A1), as previously described [17].

### 2.4. RED-Capable Strain Preparation

**Figure 1:**
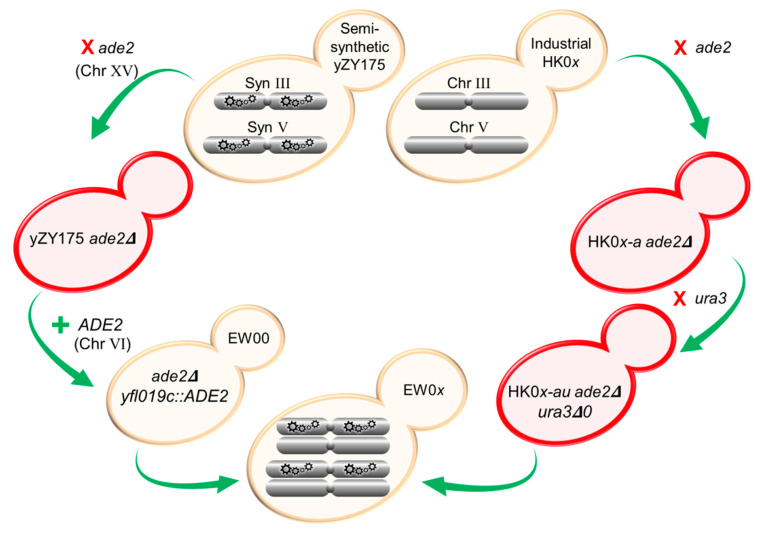
Strategy to generate RED-enabled heterozygous diploid *S. cerevisiae* strains. Native *ade2* and *ura3* were removed from industrially derived HK01–HK04 haploid strains. Native *ade2* was also deleted from Chr XV in the semi-synthetic yZY175 strain, and functional *ADE2* was re-introduced into Syn Chr VI replacing the non-essential *YFL019C* gene. In this locus, *ADE2* is flanked by the existing loxPsym sequences. Mating of the two modified parental haploids generated RED-enabled EW01–EW04 containing native (non-synthetic) chromosomes from HK01–HK04 (MATa) and synthetic chromosomes from yZY175 (MATα). Colors of yeast strains shown in this figure are representative of the color of strains following each genetic manipulation.

Strain preparation is summarized in Figure 1. To introduce RED capability into the strains intended for SCRaMbLE library generation, the native *ADE2* on chromosome XV of the haploid strains HK01–HK04 and yZY175 was replaced with a *kanMX4* marker, conferring resistance to geneticin. The *ade2::kanMX4* locus, along with ~200 bp flanking sequences, was PCR amplified from genomic DNA (extracted using the SDS/LiOAc genomic DNA extraction procedure [18]) obtained from the BY4741 *ade2Δ* strain [19] (Table 2). The *kanMX4*-containing fragment was transformed into all five haploid strains to replace and disrupt the native *ADE2* gene using the LiOAc/SS carrier DNA/PEG method [16]. Transformants (red colonies) were selected on YPD agar supplemented with 200 μg mL^−1^ G418-sulfate (Roche) and to reflect a lack of *ADE2* were designated HK01-a, HK02-a, HK03-a and HK04-a, based on the corresponding isogenic strain used.

A functional copy of *ADE2* was reintroduced into a synthetic chromosome of strain yZY175 *ade2*Δ to complement the *ade2* deletion from native Chr XV, producing white-colored colonies. The *ADE2* CDS (with ~500 bp upstream and downstream flanking sequences) was amplified from *S. cerevisiae* BY4741 using forward and reverse primers ‘ADE2 + YFL019C’ using the same PCR conditions as above (Table A1). The primers added 40 bp of flanking sequences homologous to the YFL019C locus on Chr VI. The *ADE2*-containing PCR fragment was then transformed into synthetic Chr VI of the yZY175 *ade2*Δ, disrupting the nonessential gene YFL019C, thus generating a loxPsym-flanked *ADE2*. White colonies were selected on adenine-deficient (SC^-ade^) agar and the selected isolate was named EW00. 

To allow the use of the Cre-recombinase expression plasmids containing *URA3* as a selectable marker, the native *URA3* was deleted from the HK01-a, HK02-a, HK03-a and HK04-a strains. The *ura3Δ0* locus of the *S. cerevisiae* BY4742 strain was amplified using forward and reverse ‘Ura3′ primers that annealed approximately 250 bp upstream and downstream of the native CDS (Table A1). The fragment was transformed into the four strains and transformants were selected on YPD agar supplemented with 1 g L^−1^ Thermo Scientific™ 5-FOA (Fluoroorotic acid). The absence of a functional *URA3* was subsequently confirmed by the absence of growth on media lacking uracil (SC^-ura^ agar); these strains were identified using the following convention: HK0*x*-au.

Heterozygous diploid strains EW01, EW02, EW03 and EW04 were generated by mating each of the HK01-au, HK02-au, HK03-au and HK04-au with the semi-synthetic EW00. Individual strains were grown overnight in YPD broth, inoculated into fresh YPD broth to OD_600_ 0.125 and incubated for 3 h. Cultures were then diluted to OD_600_ 0.5 and co-cultured overnight in equal proportions in YPD broth at an ambient temperature to allow mating. Cell suspensions were plated on SC^-ade^ to limit the growth of the industrial haploid colonies. Diploid colonies were identified by mating-type PCR, as described earlier. 

To study the dynamics of previously reported SCRaMbLE induction systems, facilitated by different native yeast promoters driving Cre-recombinase expression, EW01 was transformed with either pLM160, pEW_GAL1p or pEW_SCW11p and putative transformants selected on SC^-ura^ agar plates, to produce strains EW01-CLB, EW01-GAL and EW01-SCW, respectively.

**Table 2:** Summary of relevant *S. cerevisiae* strains used in this study.

Strain	Description	Genotype	Reference
yZY175	Contains synthetic chromosomes III, VI and IX-R	MATα	[20]
MH-1000	Industrial distillery yeast	MATa/MATα	[21]
Y-11878	Isolated from Jamaican cane juice	MATa/MATα	[22]
YB-428	Isolated from rum fermentation	MATa/MATα	[22]
Y-582	Isolated from dry claret wine	MATa/MATα	[22]
HK01	Progeny derived from MH1000	MATa ho::GAL1_p_—kanMX4	This study
HK02	Progeny derived from Y-11878	MATa ho::GAL1_p_—kanMX4	This study
HK03	Progeny derived from Y-582	MATa ho::GAL1_p_—kanMX4	This study
HK04	Progeny derived from YB-428	MATa ho::GAL1_p_—kanMX4	This study
HK01-au	Uracil / adenine auxotrophic HK01	MATa ura3Δ ade2Δ::kanMX4	This study
HK02-au	Uracil / adenine auxotrophic HK02	MATa ura3Δ ade2Δ::kanMX4	This study
HK03-au	Uracil / adenine auxotrophic HK03	MATa ura3Δ ade2Δ::kanMX4	This study
HK04-au	Uracil / adenine auxotrophic HK04	MATa ura3Δ ade2Δ::kanMX4	This study
EW00	RED-enabled yZY175 (haploid)	MATα ura3Δ ade2Δ::kanMX4 yfl019c::ADE2	This study
EW01	RED-enabled HK01-au X EW00	MATa/MATα ura3Δ/ura3Δ ade2Δ::kanMX4/ ade2Δ::kanMX4 YFL019C/yfl019c::ADE2	This study
EW02	RED-enabled HK02-au X EW00	MATa/MATα ura3Δ/ura3Δ ade2Δ::kanMX4/ade2Δ::kanMX4 YFL019C/yfl019c::ADE2	This study
EW03	RED-enabled HK03-au X EW00	MATa/MATα ura3Δ/ura3Δ ade2Δ::kanMX4/ade2Δ::kanMX4 YFL019C/yfl019c::ADE2	This study
EW04	RED-enabled HK04-au X EW00	MATa/MATα ura3Δ/ura3Δ ade2Δ::kanMX4/ade2Δ::kanMX4 YFL019C/yfl019c::ADE2	This study
EW01-CLB	EW01 with plasmid pLM160	yfl019c::ADE2 CLB2_p_ Cre_EBD	This study
EW01-GAL	EW01 with plasmid pEW_GAL1	yfl019c::ADE2 GAL1_p_ Cre_EBD	This study
EW01-SCW	EW01 with plasmid pEW_SCW11	yfl019c::ADE2 SCW11_p_ Cre_EBD	This study

### 2.5. Spot Assays for Fitness Evaluation

Industrial diploid strains, their haploid progeny (HK01–HK04) and RED-enabled diploid strains (EW01–EW04) were cultivated overnight in YPD after which they were inoculated into fresh YPD medium to a final optical density at 600 nm (OD_600_) of 0.125 and grown for 3–4 h. Cultures were washed twice with phosphate buffered saline. Cells were diluted to an OD_600_ of 0.5 and a 10× dilution series spotted on YPD agar plates containing either 10% *v*/*v* ethanol, 12% *v*/*v* ethanol, 1.5 M sorbitol or 25 mM dithiothreitol (DTT).

### 2.6. Ethanol Production Determination

Cultures of heterozygous strains EW01–04 and industrial diploids MH-1000, Y-11878, YB-428 and Y-582 were grown overnight in YPD. The cultures were then inoculated into 40 mL of fresh YPD with high glucose concentration (200 g L^−1^ glucose) in 50 mL falcon tubes to an OD_600_ of 0.2. A rubber stopper with S-bend airlocks filled with ~2 mL of sterile water was attached to the top of the Falcon tubes to allow for CO_2_ escape. The cultures were incubated at ambient temperature for 120 h to allow the fermentation to finish. The final ethanol concentration in culture supernatant was measured using the Megazyme ethanol assay kit (K-ETOH, Megazyme, Ireland) as per the manufacturer’s instructions.

### 2.7. SCRaMbLE

**Figure 2:**
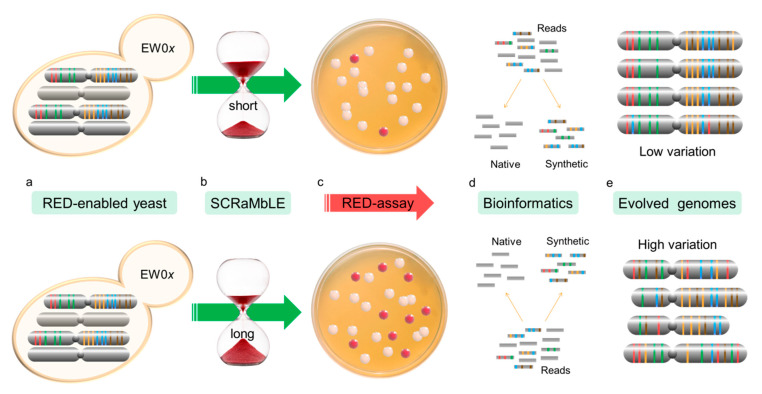
Flow diagram of the RED assay experimental procedure. RED-enabled semi-synthetic heterozygous diploid cells, containing a synthetic copy of chr III and VI, were engineered to turn red due to loxP-mediated loss of *ADE2* (**a**). Cre-recombinase expression was induced for 2, 4 and 6 h to allow increased genomic rearrangement through SCRaMbLE (**b**). The frequency of *ADE2* loss was determined based on the appearance of yeast colonies with red pigment (**c**). Randomly selected colonies were sequenced using short-read NGS technology. Custom scripts were developed to filter out all reads not originating from the synthetic genomes (**d**). SCRaMbLEd chromosomes were assembled and assessed for gene loss (**e**).

The RED-assay workflow is shown in Figure 2. Strain EW01-GAL was cultivated overnight at 30 °C in YPD broth, then inoculated into fresh YPD to an OD_600_ of 0.2 and cultivated for 3–4 h. To induce SCRaMbLE, cells were washed twice with ddH_2_O and inoculated into fresh YP galactose broth (20 g L^−1^ galactose) supplemented with 1 µM β-estradiol at an OD_600_ of 0.2. In addition to this, EW01-GAL cultures were also prepared in YP galactose without β-estradiol and YPD with and without β-estradiol. Cells were incubated with 200 rpm shaking at 30 °C and samples were taken at 0, 2, 4 and 6 h, washed twice with ddH_2_O and plated on YPD agar. Plates were incubated for 2–3 days at 30 °C before colony numbers and colors (red or white) were recorded. A random selection of eight red and eight white colonies from each time point was analyzed by PCR to indicate the presence or absence of chromosome arms. One pair of primers was used for each chromosome arm of the native and synthetic Chr III and VI (Table A1).

### 2.8. Genome Sequencing

Twelve white post-SCRaMbLE EW01-GAL colonies from each time point were randomly selected from the cultures that had been plated onto YPD agar after 2, 4 and 6 h of induction. In addition, two red colonies from each time point were selected. Each selected colony was grown overnight in YPD broth and genomic DNA was extracted from each culture using the Thermo Scientific™ Yeast DNA Extraction Kit as per the manufacturer’s instructions. Paired-end whole genome sequencing was carried out at the Beijing Genome Institute (BGI), Beijing, China using the Illumina sequencing technology BGISEQ PE100 at 30× coverage. The length of each sequencing read after adapter trimming was 100 bp.

### 2.9. Detection of SCRaMbLE Events

HK01, EW00, EW01 and 48 SCRaMbLEd *S. cerevisiae* strains were sequenced. The determination of SCRaMbLE events in heterozygous diploid strains such as EW01 is technically challenging given the high sequence similarity between corresponding synthetic and ‘native’ DNA. As such, it was important to remove reads originating from ‘native’ DNA from the read pool as they cannot be SCRaMbLEd and could skew the interpretation of the results. A custom bioinformatics pipeline was therefore developed to remove these sequencing. Briefly, the script (supplementary material) used Bowtie 2 [23] to map all reads to two reference sequences—Syn III and VI of the semi-synthetic strain EW00, and Chr III and VI of the MH-1000-derived HK01. Using filtering strategies, reads were isolated that satisfied two requirements—firstly, they mapped with 100% similarity to Syn III or VI of EW00, and secondly, they did not map with 100% similarity to Chr III and VI of HK01. For unpaired reads recovered this way in the read pool, their read partner was recovered as well. These reads therefore contained sequences exclusively generated from the synthetic chromosomes and were used to infer subsequent deletion events. For convenience in subsequent analyses, the scripts were designed to generate a standardized FASTA formatted file. The bioinformatics procedure is visually represented in Figure A4. For quality control, an additional output of the script is a log file containing information from each step of the script, including, for example, the total number of reads and how many mapped to each reference (with and without 100% similarity). The filtered read pool was imported into Geneious Prime 2020.0.4 (https://www.geneious.com) and mapped to a consensus sequence of Syn Chr III and VI. The missing CDS annotations from each strain were compiled, enumerated and visualized in a heat map using GraphPad Prism version 8.01 for Windows, La Jolla California USA, www.graphpad.com. 

## 3. Results

### 3.1. Heterozygous Diploid S. cerevisiae for Rapid Evolution Detection SCRaMbLE

To enable RED in *S. cerevisiae* strains, at least one functional copy of *ADE2* should be present in the genome, flanked by loxP-recombination site*s*. This was achieved by the introduction of an *ADE2* gene cassette into the loxP-flanked *YFL019C* locus of the semi-synthetic yZY175 *ade2*Δ strain. This RED-facilitator strain enabled the generation of four semi-synthetic strains through a simple mating procedure with industrially relevant, *ADE2*-deficient yeast strains. All four RED-enabled heterozygous diploid strains (EW01–EW04) had consistent white-cream colored colonies on YPD agar plates, with no spontaneous red colored colonies or sectoring detected at any stage. The presence of unique, locus-specific PCR-tags (a feature of the synthetic chromosomes [2]) implied the presence of the native and synthetic chromosomes III and VI, in all four RED-enabled heterozygous diploids. Illumina genome sequencing confirmed the presence of intact synthetic chromosomes III and VI, in addition to its native counterparts. No aberrant gene or chromosomal copy-numbers were detected.

### 3.2. General Stress-Resistance and Ethanol Production of Heterozygous Diploid Strains

**Figure 3:**
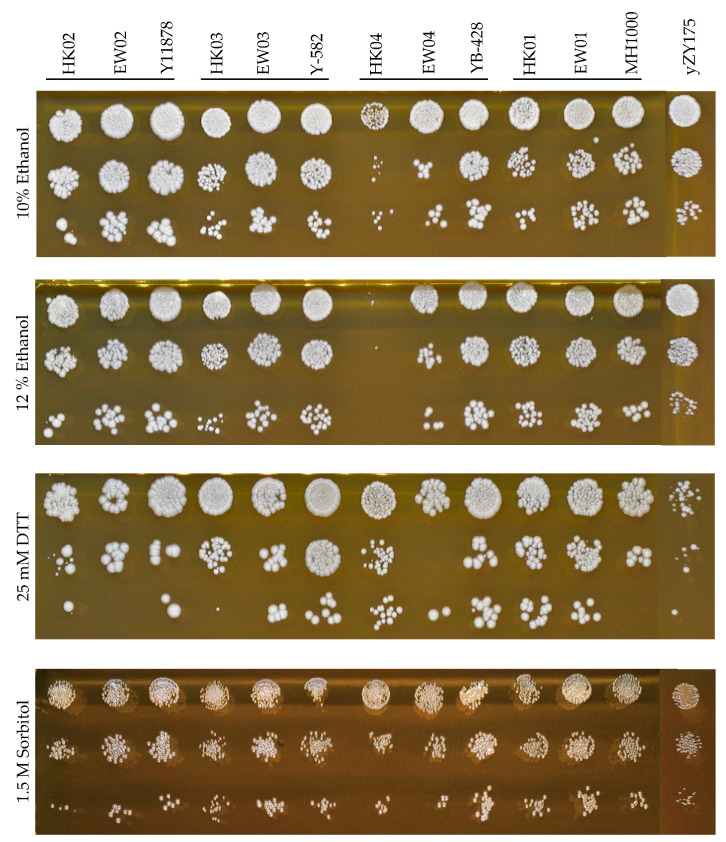
Fitness assays of generated heterologous diploid strains EW01-04, their parental haploids (HK01–04 and yZY175) and the corresponding diploid strains (industrial diploids). Spot assays were performed on YPD containing 10–12% (*v*/*v*) ethanol, 25 mM dithiothreitol (DTT) and 1.5 M sorbitol incubated for 72 h.

In general, the semi-synthetic heterozygous diploids shared the combined properties of both synthetic and industrial yeast backgrounds. In addition to the ability to undergo rapid genome evolution, facilitated by the presence of many loxP sites on the synthetic chromosomes III and VI, the fitness of these heterologous diploids was similar to that of their respective parental diploid strains under various stress conditions. The novel diploid strains displayed high tolerance to osmotic stress (up to 1.5 M sorbitol), reducing conditions (up to 25 mM DTT) and presence of alcohol to up to 12% (*v*/*v*) ethanol, respectively (Figure 3). It is noted that the growth of the haploid industrial parent strains HK03 and HK04 was inhibited under ethanol and reducing conditions; however, the tolerance phenotypes were recovered in their corresponding heterozygous diploid strains.

**Figure 4:**
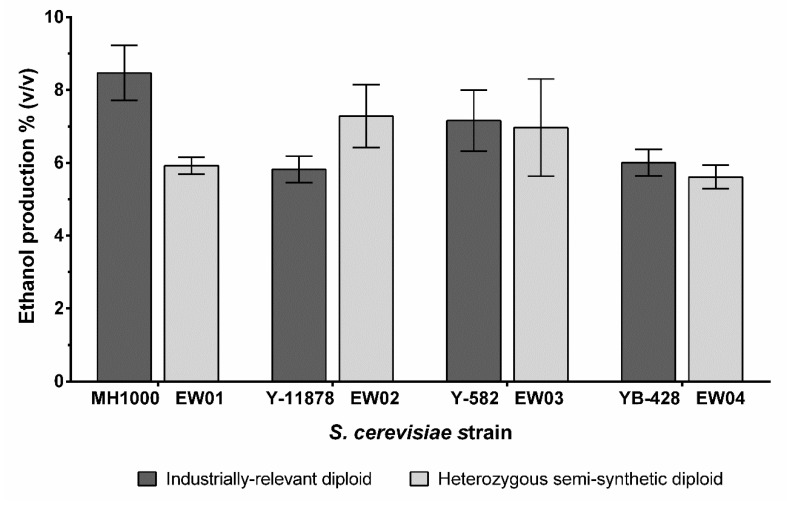
Ethanol production of *S. cerevisiae* industrially relevant diploids (MH1000, Y-11878, Y-582 and YB-428) and semi-synthetic heterozygous diploids (EW01-EW04). Ethanol concentration was measured after cultivation in YPD with high glucose concentration (20 g L^–l^ glucose) at 30 °C for 120 h. There was no difference in ethanol production from semi-synthetic strains compared to the corresponding industrial diploids, except for the EW01 strain, compared to the parental MH1000 strain.

In addition to the general stress tolerance of yeast strains, ethanol production is an important trait for many industrial *S. cerevisiae* strains; as such, the end-point ethanol production of the generated diploids was assessed. The semi-synthetic nature of the EW03 and EW04 strains did not affect their final ethanol yields compared to their corresponding diploid industrial parent strains (Figure 4). EW01 produced significantly less ethanol than its industrial diploid parent (*p* < 0.02), at just under 6% *v*/*v*, compared with the ~8.5% *v*/*v* achieved by the MH1000 strain under our culturing conditions. Interestingly, the heterozygous EW02 on average produced up to 1% *v*/*v* more ethanol than its corresponding industrial diploid parent, Y-11878.

### 3.3. SCRaMbLE Induction and Rapid Evolution Detection

**Figure A1:**
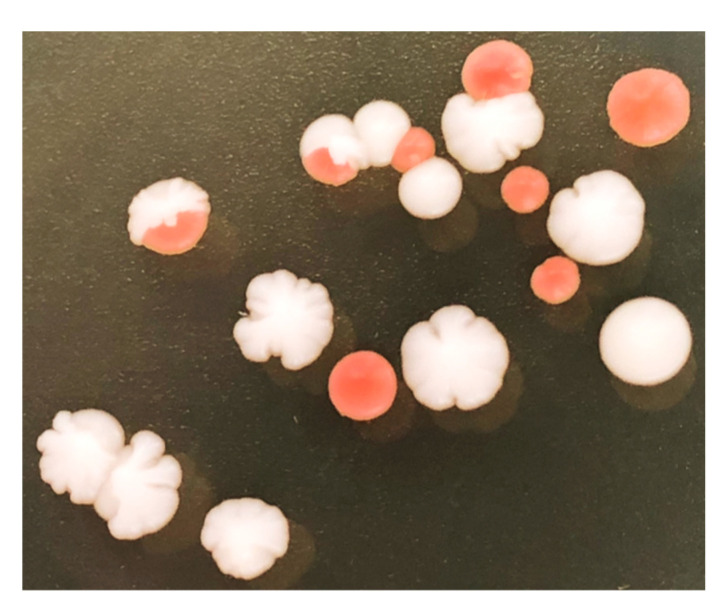
Heterozygous diploid *S. cerevisiae* EW01-GAL colonies after SCRaMbLE. A clear difference between red and white colonies is observed.

The RED-enabled strains developed in this study provide a viability-independent visual output which reflects the scale of genome rearrangement within a post-SCRaMbLE yeast population. Based on the visibly red colonies produced by *ade2* mutants (due to the accumulation of red-pigment [24]), our RED assay generates a visual estimation of the frequency of Cre-induced recombination, and the subsequent gene loss of the loxP-flanked *ADE2* cassette (Figure A1). 

**Figure 5:**
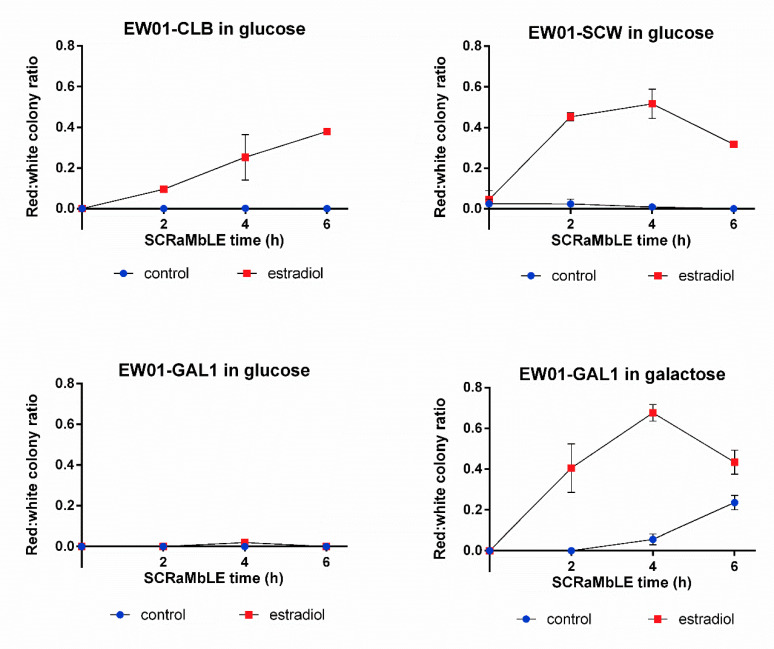
Red:white ratio of *S. cerevisiae* heterozygous diploid EW01 colonies following SCRaMbLE for up to six hours. Three strains were evaluated (EW01-CLB, EW01-SCW and EW01-GAL) that contained Cre expression plasmids with *CRE* under the transcriptional control of promoters *CLB2*p, *SCW11*p and *GAL1*p, respectively. Cultures were supplemented with 1 μM estradiol to induce SCRaMbLEing, while the control cultures received no estradiol. EW01-GAL was cultivated in both inducing (galactose in the absence of glucose) or non-inducing (glucose-containing) conditions. At two-hour time points from 0 h, samples of cells were taken, washed and plated onto YPD agar. The color of colonies was recorded after 72 h.

Using the RED-assay, we compared the SCRaMbLE induction dynamics of three promoters, previously used for Cre_EBD expression. The promoters used to drive Cre_EBD expression were the M/G1 cell-cycle phase-activated *CLB2* promoter [25,26], the daughter cell-specific activated *SCW11* promoter [27] and the galactose-inducible *GAL1* promoter [28]. In addition to the promoter-dependent expression patterns of the recombinase, estradiol is required for SCRaMbLE induction to allow the nuclear-localization of the estradiol binding domain-linked Cre-recombinase [6]. As unintended genome rearrangement can lead to preemptive genome instability, the basal recombination rate of the EW01 strains, harboring either the pEW_CLB, pEW_SCW11 or the pEW_GAL plasmid, were evaluated with RED (Figure 5). In the absence of estradiol, no red colonies were detected at any evaluated time point for the strains harboring the pEW_CLB or pEW_GAL plasmids when galactose was absent (Figure 5). Surprisingly, red colonies were observed in the EW01 strain containing the pEW_SCW plasmid, where the number of red colonies remained below 5% of the population when no estradiol was present (Figure 5). In the presence of galactose, but no estradiol, the EW01_GAL strain showed a continuous increase in red colonies over time, reaching significantly higher red colony formation frequencies compared to glucose-grown cells at 4 h and 6 h of growth (Figure 5). Up to a quarter of the population was red after 6 h of galactose growth.

With the exception of the glucose-grown EW01-GAL strain, the rapid generation of red colonies was observed after 2 h of estradiol addition (Figure 5), with more than 65% of the post-SCRaMbLE population being red in the EW01-GAL (grown in galactose) strain after 4 h of estradiol exposure (Figure 5). There was a gradual increase in red colony frequencies for all strains up to the 4 h time point, after which the EW01-SCW and EW01-GAL (grown in galactose) strains had reductions in the ratio of red colonies at 6 h after estradiol addition. 

### 3.4. Quantitation of SCRaMbLE Events

The impact of SCRaMbLE on a genomic level was subsequently investigated using the EW01-GAL strain, since it had the greatest versatility for Cre-induction options and demonstrated the effective suppression of SCRaMbLE in glucose-containing media. Twelve white colonies and two red colonies were randomly selected from each time point after estradiol addition (2, 4 and 6 h) in the galactose–grown EW01-GAL strain (Figure 5). At each time point, the total number of colonies obtained was 63, 141 and 200, respectively. The genome of each colony was sequenced and the synthetic portion analyzed with custom scripts (developed in this study) to evaluate the level of SCRaMbLE that occurred. The degree of genome rearrangement was based on the number of genes lost per genome in each SCRaMbLEd colony.

**Figure A2:**
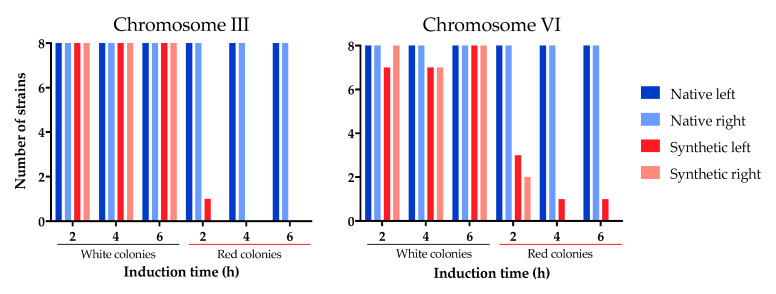
Detection of PCR markers on the native and synthetic chromosomes of SCRaMbLEd EW01-GAL strains. Red colonies displayed a high frequency of synthetic chromosome loss, while maintaining native chromosome integrity.

The analysis of sequencing data from red-pigmented colonies revealed that, in general, large amounts of genetic material were lost on both synthetic chromosomes. At least 50%, and up to 78%, of all CDSs had been deleted in all analyzed red strains (data not shown). This phenomenon of large amounts of DNA loss in red colonies was supported by the absence of synthetic chromosome-specific PCR products obtained from a larger set of 24 red and 24 white colonies (Figure A2). The absence of PCR amplification products from targets on both chromosome arms might suggest a high frequency of complete loss of synthetic chromosomes in red colonies. Interestingly, red colonies obtained after two hours of SCRaMbLE displayed extensive gene loss, similar to those analyzed at the 4 and 6 h time points.

**Figure 6:**
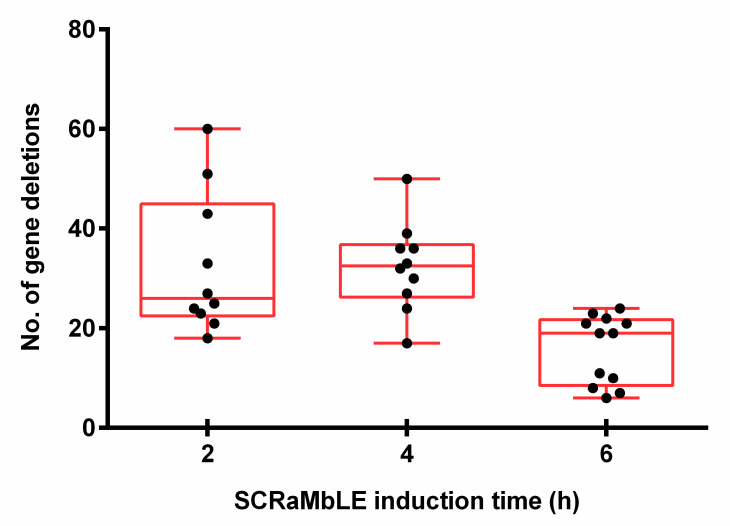
The number of deleted genes in white EW01_GAL colonies obtained after two, four or six hours of estradiol exposure. Each black dot represents an individually sequenced SCRaMbLEd EW01_GAL colony.

**Figure A3:**
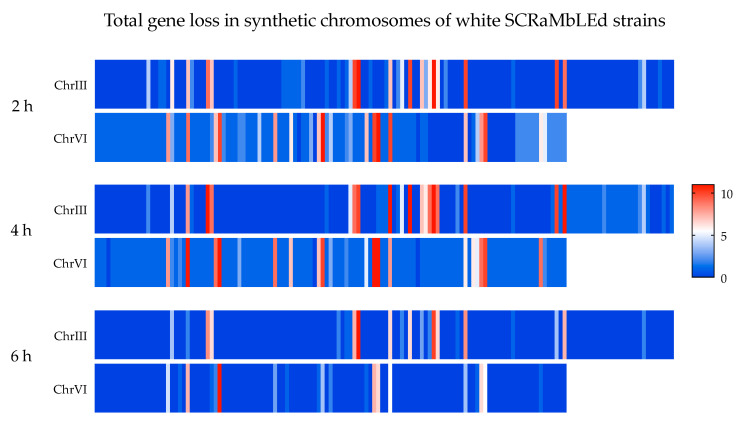
Heat maps of gene loss frequencies in three sets of randomly selected white SCRaMbLEd EW01-GAL. Each segment represents a coding DNA sequence (CDS) on synthetic chromosomes III and VI, while the color depicts the total number of CDS lost by SCRaMbLEing; blue segments depict sites of minimal deletion while red represents the most frequent deletion sites.

**Figure A4:**
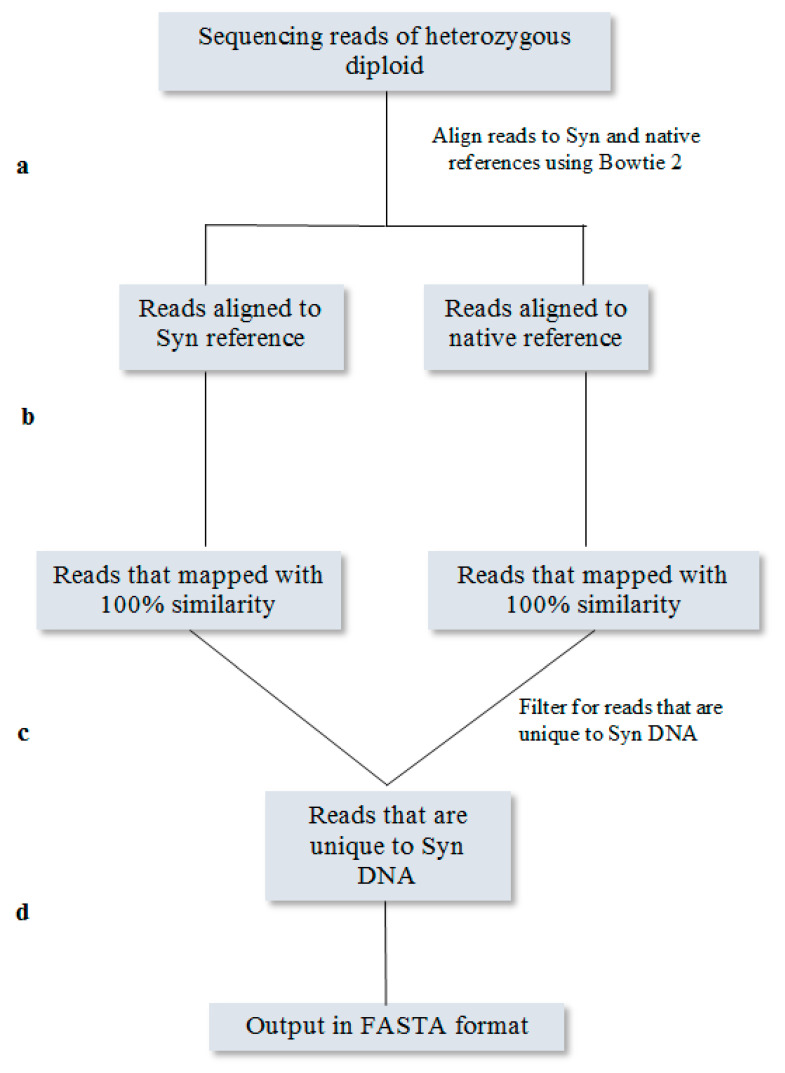
Summary of bioinformatics pipeline to filter sequencing reads of SCRaMbLEd heterozygous diploid *S. cerevisiae*. Reads are mapped to reference sequences from both parental chromosomes (**a**) and are filtered by reads that were a 100% match to the reference (**b**). Pools of reads are compared and reads that uniquely match to the synthetic reference are separated (**c**). The final read pool is converted into FASTQ for convenient direct use in other software (**d**).

With the exclusion of two outlier strains, which had lost the majority of synthetic chromosome VI, a significant variation in gene-loss frequencies was observed between the white colonies evaluated at each time point, with 7–60 gene deletions per strain (Figure 6). On average, strains had 26 deletions after 2 h of induction, which increased to 33 at 4 h of estradiol introduction. In accordance with the RED assay, the average number of gene deletions decreased to 19 per strain for the 6 h time point. A genomic heat map (Figure A3), showing the frequency of gene loss across synthetic chromosomes III and VI, revealed a non-random distribution of gene deletion events over the length of both synthetic chromosomes. 

## 4. Discussion

SCRaMbLE is a novel genome evolution system associated with the synthetic Sc2.0 strains, allowing the combinatorial deletion, duplication and translocation of multiple genes at a time. Not only is SCRaMbLE a valuable tool for studying epistatic interactions between genes, it has also been demonstrated as a useful approach to generate large libraries of novel strains with improved industrial phenotypes [7,12,15,29]. However, this indiscriminatory rearrangement of functional genomic units frequently results in inviable or unfit phenotypes due to the loss of essential genes or the disruption of fitness-related metabolic pathways. To harness SCRaMbLE for the development of next generation industrial strains, precise control is required to limit the loss of promising strains due to excessive gene loss. Currently, strategies to indicate the degree of genomic SCRaMbLEing are based on the evaluation of cell viability and time-consuming whole genome sequencing analysis [8], though both approaches are of limited use in industrial yeast strains, which are predominantly diploid. Here, a simple method called rapid evolution detection (RED) was developed for use in semi-synthetic heterozygous yeast strains. RED allows the qualitative detection of the genomic rearrangements that occurred within a SCRaMbLEd population through the generation of visually distinct red-pigmented yeast colonies.

SCRaMbLE-mediated genomic rearrangements occur exclusively in synthetic DNA at the gene-flanking loxP sites. Here, a RED-enabled semi-synthetic haploid strain (EW00) was constructed and used as a modular add-on to generate semi-synthetic heterozygous yeast strains with industrial backgrounds. Similar to other strain-breeding reports [30], the four semi-synthetic diploid strains generated here displayed a minimal loss of beneficial phenotypes (and even heterosis). Although a limited number of phenotypes were evaluated here, our results suggest that the heterozygous synthetic DNA had a low impact on the general fitness of the resulting strains, and that it is possible to introduce SCRaMbLE-capability into any industrial *S. cerevisiae* strain.

To restrict excessive genome rearrangement, SCRaMbLE was originally designed with an inducible control mechanism, whereby Cre-recombinase would only be expressed in newly formed daughter cells and only activated in the presence of estradiol [2]. This was achieved by fusing the Cre-recombinase to the murine estradiol-binding domain (EBD), which sequesters Cre-recombinase in the cytosol. The controlled addition of estradiol to the culture medium facilitates the movement of Cre-EBD into the nucleus, where Cre recombinase is able to act upon the loxP sequences of the synthetic genome [2]. However, reports from our group and other Yeast 2.0 consortium members have suggested that SCRaMbLE might occur even in the absence of estradiol. RED revealed low, but detectable, *ADE2* deletion events for strains harboring *CLB2*_p_ and *SCW11*_p_ Cre-EBD expression plasmids in the absence of estradiol. Even at a low frequency, this could lead to unintended gene loss or even the loss of whole chromosomes without Cre-induction [6,15], and could affect the long-term stability of strains. Additional evidence of this leakiness was observed in RED-enabled strains grown on agar plates (in the absence of estradiol), with the infrequent appearance of the red sectoring (Figure A1) of otherwise white colonies—a strong indicator of genome instability [31]. The unintended SCRaMbLE initiation in the absence of estradiol is likely to be Cre-EBD concentration-dependent, as the strong induction of the recombinase expression from the *GAL1* promoter surpassed the cytosolic sequestering ability of the estrogen-binding domain. This observation shows the versatility of the *GAL1*_p_ expression system for Cre-induction, by providing options for a stepwise adjustment in SCRaMbLE strength, in addition to the effective suppression of SCRaMbLE in glucose-containing media. 

The custom bioinformatic pipeline developed in this study was able to effectively differentiate short DNA reads originating from the synthetic portion of heterozygous *S. cerevisiae* strains, allowing the enumeration of gene deletion events after SCRaMbLE. The genome sequencing data supported the visual RED assay results. Using this simple method to visually report on the frequency of genomic rearrangements in a post-SCRaMbLE population, we were able to detect the leakiness of several Cre-expression systems and establish the induction timeframes for optimal SCRaMbLEing rates. Our results also demonstrated the importance of selecting appropriate sampling times, as prolonged SCRaMbLE induction could ultimately reduce the frequency of genomic rearrangement within the population. One reason for this observation could be linked to the loss of cell viability due to the increased chance of essential gene loss and/or the excessive disruption of cellular metabolism over extended periods of SCRaMbLE. It is thus conceivable that cells with fewer rearrangements or those which have escaped the influence of Cre-recombination (due to plasmid loss or mutations) would have a competitive advantage over cells with rapidly changing genomes.

Assuming an equal chance of recombination at any given loxP site, SCRaMbLEd synthetic haploid strains would have lost on average seven to eight genes, upon reaching 90% cell lethality due to essential gene loss. This value was in alignment with previous reports for SCRaMbLEd haploid strains that had up to eight gene deletions per strain [6]. In the heterozygous strains evaluated in this study, SCRaMbLEd populations were generated with, on average, 33 gene deletions per strain. Considering that some strains had up to 60 gene deletions, the opportunity to obtain highly modified genomes is significantly enhanced by the higher viability of these strains. SCRaMbLEing in heterozygous diploid strains has been established as a powerful tool to generate novel phenotypes (including in interspecies diploids [9]), but whether this increased genomic diversity of SCRaMbLEd heterozygous strains outweighs the potential masking effects of the native chromosome remains to be determined. 

In conclusion, RED was developed as a modular system to monitor and report on the occurrence and level of SCRaMbLEing in a population that provides information visually without the need to sequence strains. Heterozygous diploids were developed by combining industrially relevant haploids with a RED-enabled strain containing synthetic DNA, and were shown to be generally as fit as their industrial parent. Through the breeding strategy used here, or protoplast fusions, synthetic chromosomes can be introduced to any industrial *S. cerevisiae* strain and be RED-enabled. Furthermore, RED proved a valuable resource to rapidly evaluate various SCRaMbLE induction systems and induction optimizations. In future studies, RED could be harnessed to determine the effects of different growth conditions or strain backgrounds on the magnitude of SCRaMbLE in a population, or assist in future genome minimization efforts [2].

## Figures and Tables

**Table 1:** Cre-recombinase expression plasmids.

Plasmid Name	Relevant Genotype	References
pHK300-HO	*ho::GAL1_p_ –kanMX4*	[14]
pLM006	*HIS3,CEN6*, *SCW11_p_* Cre_EBD	[9,15]
pEW_CLB	*URA3*, *CEN6*, *CLB2_p_* Cre_EBD	aka pLM160 [9,15]
pEW_GAL	*URA3*, *CEN6*, *GAL1_p_* Cre_EBD	This study
pEW_SCW	*URA3,CEN6*, *SCW11_p_* Cre_EBD	This study

## Supplementary Materials

The shell scripts that was used to separate Synthetic Yeast Illumina DNA reads from a heterologous diploid strains can be accessed at https://github.com/HeinrichKroukamp/Wightman-et-al-2020-script.

## Appendix A

**Table A1:** Primers used in this study.

Primer Name	Forward
HO_ampl-F	TCACGGCTAACTCTTACGTTATG
HO_ampl-R	GTATGTACCAGAAGCACGTGAAG
HO_conf-F	ATGCTTTCTGAAAACACGACTATTC
HO_conf-R	ACAGCATCAAACTGTAAGATTCCG
Cre-F+PacI	CTACTTAATTAAATGTCCAATTTACTGACC
Cre-CassetteR	GAATTCGATATCAAGCTTATCGA
ADE2 F	AAGCCGAGAATTTTGTAACACC
ADE2 R	TCCTCGGTTCTGCATTGAGC
ADE2 + YFL019C F	CTTTGCTCTATATTCGAGTCAGGCTCCACCAGTCATTTCGTCGCCCAATGTGTCCATCTG
ADE2 + YFL019C R	ATCATAAAAACCTTACGCCATCTGTGGGACTCGTCATAGGACGAGCCGTTGATGAGGTTA
Ura3 F	GCGAGGCATATTTATGGTGAAG
Ura3 R	TGGAACTCTTGTTGTTCTTTGG
MatA-F	ACTCCACTTCAAGTAAGAGTTTG
MatAlpha-F	GCACGGAATATGGGACTACTTCG
MatLocus-R	AGTCACATCAAGATCGTTTATGG
NatIII left F	TTAGGGACGCATCTGTGAACTC
NatIII & SYNIII left R	GTTTGGAGACTTTGGATCATCC
NatIII right F	CTCTTCGGGGTACTCTTTACCT
NatIII & SYNIII right R	GTTGAAGTCACATCGTCACAGT
NatVI left F	CAGGATGTACTGATAGGACGTT
NatVI left R	GTAGCATACGCACTCCGAAC
NatVI Right F	CCTTGCTAGCATCCAAACTTTC
NatVI Right R	GATGGACTGGTGACGCAAG
SynIII left F	TCCGAGATGCTAGCGTTAATAG
SynIII right F	AGCAGCGGTTATAGCTTGCCA
SynVI right	CAAGACTGGGTGAACGCTCAA
SynVI right	GCTTTGGCCTCTATTCAAACCT
